# Effect of commercial vaginal products on the growth of uropathogenic and commensal vaginal bacteria

**DOI:** 10.1038/s41598-020-63652-x

**Published:** 2020-05-06

**Authors:** Kristin J. Hung, Patricia L. Hudson, Agnes Bergerat, Helai Hesham, Namit Choksi, Caroline Mitchell

**Affiliations:** 1 Vincent Obstetrics and Gynecology Department, Division of Female Pelvic Medicine and Reconstructive Surgery, Massachusetts General Hospital, Boston, MA United States; 2 Department of Obstetrics, Gynecology & Reproductive Biology, Harvard Medical School, Boston, MA United States; 3 Vincent Center for Reproductive Biology, Vincent Obstetrics and Gynecology Department, Massachusetts General Hospital, Harvard Medical School, Boston, MA United States

**Keywords:** Bacteria, Urology

## Abstract

Half of postmenopausal women experience genitourinary syndrome of menopause, for which many use lubricating vaginal products. The effect of vaginal products on uropathogenic and commensal vaginal bacteria is poorly understood. We evaluated the effect of five common vaginal products (KY Jelly, Replens Silky Smooth lubricant, coconut oil, Replens Long-Lasting moisturizer or Trimo-San) on growth and viability of *Escherichia coli* and *Lactobacillus crispatus*. Bacteria were co-cultured products alone and in the presence of both vaginal epithelial cells and selected products. Bacterial growth was compared between conditions using an unpaired t-test or ANOVA, as appropriate. All products except for coconut oil significantly inhibited growth of laboratory and clinical strains of *Escherichia coli* (p < 0.02). Only two products (Replens Long-Lasting moisturizer and Trimo-San) significantly inhibited growth of *Lactobacillus crispatus* (p < 0.01), while the product Replens Silky Smooth stimulated growth (p < 0.01). Co-culture of selected products in the presence of vaginal epithelial cells eliminated the inhibitory effects of the products on E. coli. In conclusion, *in vitro* exposure to vaginal moisturizing and lubricating products inhibited growth of *Escherichia coli*, though the inhibition was mitigated by the presence of vaginal epithelial cells. *Lactobacillus crispatus* demonstrated less growth inhibition than *Escherichia coli*.

## Introduction

Urinary tract infections (UTIs) are among the five most commonly diagnosed problems in the ambulatory urologic setting, and over 80% occur in women^1^. Up to 70% of women will experience at least one UTI in their lifetime, and one third to one half will experience a recurrence^2^. An estimated 10% of women over 60 are diagnosed with recurrent UTIs (rUTI), which are becoming increasingly difficult to treat as antibiotic resistance limits therapeutic and preventive options^3,4^. Over 80% of community-acquired UTIs are caused by *Escherichia coli*^5^, and recurrent UTIs are strongly linked to vaginal *E. coli* colonization^6^.

*Lactobacillus* spp. dominance of the vaginal microbiota is an important factor in maintaining urogenital health. Decreased vaginal *Lactobacillus* spp. colonization is associated with increased UTI risk, and a lack of hydrogen peroxide (H_2_O_2_)-producing lactobacilli has been associated with vaginal *E. coli* colonization in women with rUTI^7,8^. While H_2_O_2_ is no longer thought to be the mechanism of beneficial effects, the ability to produce H_2_O_2_ *in vitro* is a marker of species (e.g. *L. crispatus*) and strains that are more likely to be beneficial than non-producing species and strains (e.g. *L. iners*). A vaginal *L. crispatus* probiotic was found to reduce recurrent UTI in women who established high quantity colonization^9^, indicating a benefit of vaginal colonization with *Lactobacillus* spp. in women with recurrent UTIs.

The use of over-the-counter vaginal lubricants and moisturizers for vulvovaginal dryness or sexual activity is common, especially among postmenopausal women^10^. These products commonly contain antimicrobial preservatives such as parabens, glycerine, or chlorhexidine^11^, which may affect the vaginal microbiota and thus urogenital health. Products with and without microbicidal effect may cause inflammation, as one limited study of intravaginal nonoxyl-9 (an ingredient in spermicide and a commonly used cytotoxic control) and universal placebo gel (isotonic) found that both have inflammatory effects on cells of the cervix and endometrium^12^. Additionally, individual ingredients commonly used in vaginal products have been found to alter the electrical resistance and morphology of ectocervical tissue^13^, and hyperosmolar lubricants have been demonstrated to be cytotoxic to vaginal epithelial cells, some of them also altering inflammatory mediators^14^. While these products have been shown to negatively impact cells of the female reproductive tract, little is known about how vaginal lubricants impact potentially protective *Lactobacillus* spp. or strains of uropathogenic *E. coli*. The aim of this study was to better understand the interaction between various vaginal products, commensal vaginal bacteria and a significant uropathogen. We evaluated the *in vitro* effect of lubricants, moisturizers and a pH balancing gel on the growth of clinical and lab strains of *E. coli* and a common vaginal *Lactobacillus* spp.

## Results

### *E. coli* growth

**Figure 1:**
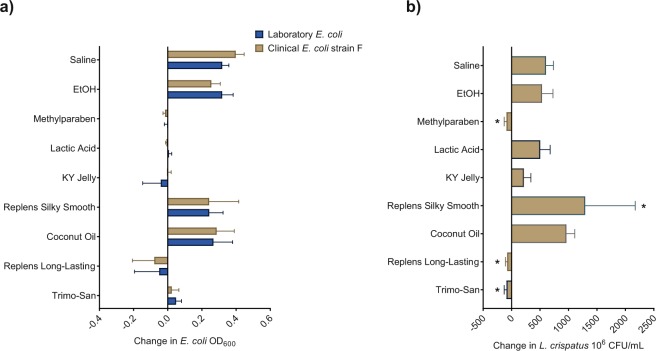
Change in bacterial growth with exposure to vaginal products. (**a**) Final change in growth, as measured by OD600, for laboratory strain of *E. coli* is similar to that for representative strain (F) of clinical *E. coli* across all test conditions (p > 0.05). (**b**) Final change in growth of *L. crispatus*, as measured in CFU (simple co-culture, without vaginal epithelial cells), demonstrating inhibition by Replens Long-Lasting moisturizer and Trimo-San (p < 0.01). *L. crispatus* growth was stimulated by Replens Silky Smooth (p < 0.01). As expected, methylparaben killed *L. crispatus* (p < 0.01), and lactic acid did not have a significant effect compared with control.

To assess the ability of products to inhibit *E. coli* growth, a mixture of *E. coli* broth culture, nutrient media and product (neat, or diluted 1:2 in sterile phosphate buffered saline (PBS) to facilitate pipetting for Trimo-San and Replens Long-Lasting) was incubated at 37 °C and compared to the addition of nutrient media alone. A 50uL volume of product was used in a 150uL total experimental volume, effectively a 1:3 or 1:6 dilution, which is comparable to what might be expected *in vivo*. Over six hours of co-culture, the growth of one laboratory and 6 clinical strains of *E. coli* were significantly inhibited in coculture with all vaginal products tested (Table 1) except coconut oil (p < 0.02 for all), with an effect seen as soon as 2 hours (Fig. 1a and Supplemental Fig. 1). Lactic acid and methylparaben both significantly inhibited growth of all *E. coli* strains (p < 0.01). Laboratory and clinical strains overall showed similar inhibition patterns, with the exception of clinical strain D, which demonstrated relatively poor growth overall (Supplemental Fig. 1). After six hours of co-culture, both the laboratory strain and a representative clinical strain of *E. coli* (from a postmenopausal woman with cystitis) were similarly impacted by the tested products (Fig. 1a).

**Table 1:** Ingredients and pH of the reagents used.

Product or Control	pH	Ingredients
Phosphate buffered saline	7.41	monobasic potassium phosphate, sodium chloride, and dibasic sodium phosphate
Ethanol	7.51	70% EtOH diluted 1:10 with PBS
Methylparaben	6.75	10% methylparaben reconstituted in 70% EtOH, diluted 1:10 with PBS
Lactic acid	4.01	50 mM racemic (DL-isomers) lactic acid diluted with PBS
KY Jelly lubricant	4.18	Water, glycerin, hydroxyethylcellulose, chlorhexidine gluconate, gluconolactone, methylparaben, sodium hydroxide
Replens Silky Smooth lubricant	5.95	Dimethicone (polydimethylsiloxane), dimethiconol
Coconut oil lubricant	6.07	Unrefined virgin coconut oil
Replens Long-Lasting moisturizer	3.18	Purified water, glycerin, mineral oil, polycarbophil, carbomer homopolymer type B, hydrogenated palm oil glyceride, methylparaben, sorbic acid, sodium hydroxide
Trimo-San pH balancer	4.0	Hydroxyquinoline sulfate 0.025%, sodium lauryl sulfate 0.01%, triethanolamine, glycerin, carbomer, citric acid, sodium citrate, methylparaben, perfume, antifoam emulsion

**Figure 2:**
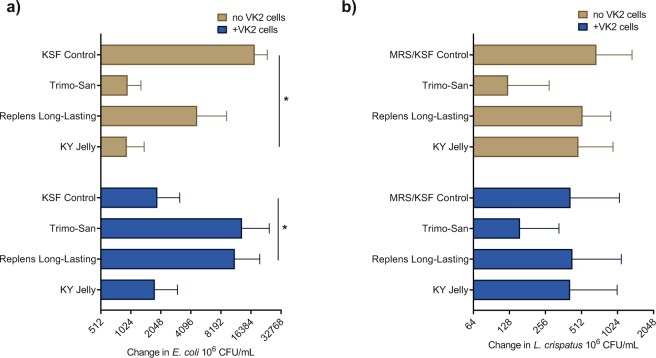
Change in bacterial growth with exposure to vaginal products in the presence of vaginal cells. (**a**) Final change in growth of *E. coli*, as measured in CFU (in 1:100 diluted product and KSF cell culture media as control), in the absence and presence of vaginal epithelial cells, demonstrating growth in Trimo-San and Replens Long-Lasting moisturizer in the presence of human cells (p < 0.05). (**b**) Final change in growth of *L. crispatus*, as measured in CFU (in 1:100 diluted product and MRS/KSF cell culture media as control), demonstrating a lack of inhibition by KY Jelly, Replens Long-Lasting moisturizer and Trimo-San, regardless of the presence of human vaginal epithelial cells (p > 0.05).

To test the observed interactions in a more high-fidelity model, the vaginal products with the most significant inhibitory effects in simple co-culture were then co-cultured with bacteria in the presence of an immortalized vaginal epithelial cell line (VK2, ATCC, VA) grown in keratinocyte serum-free (KSF) media. A 1:100 dilution of products was used to have a uniform dilution that also maintained viability of human cells with all products, and was chosen based on prior publications and preliminary experiments (Supplementary Fig. 2). When exposed to a 1:100 dilution of KY Jelly, Replens Long-Lasting moisturizer, or Trimo-San in KSF, the laboratory strain of *E. coli* was inhibited compared to a KSF media control (p = 0.01, Fig. 2a). However, in the presence of human vaginal epithelial cells, *E. coli* demonstrated no inhibition in the presence of KY Jelly, and increased growth with Replens Long-Lasting and Trimo-San compared to control (p < 0.05, Fig. 2a). Cytotoxicity to VK2 cells among the products versus no product control was not significantly different (Supplemental Fig. 2).

### *Lactobacillus* spp. growth

Over 12 hours of co-culture, *L. crispatus* growth in MRS (deMan, Rogosa and Sharpe) broth was inhibited by Replens Long-Lasting moisturizer and Trimo-San (p < 0.01), but not by KY Jelly or coconut oil (p > 0.05, Fig. 1b). *L. crispatus* growth was stimulated by Replens Silky Smooth (p < 0.01). As expected, methylparaben killed *L. crispatus* (p < 0.01), and lactic acid did not have a significant effect compared with control.

We then repeated these experiments in the presence of the immortalized human vaginal epithelial cells, using the same 1:100 dilution described above. When exposed to a 1:100 dilution of KY Jelly, Replens Long-Lasting moisturizer, or Trimo-San in KSF media, the growth of *L. crispatus* was no longer inhibited (p > 0.05, Fig. 2b). This lack of inhibition persisted in the presence of human vaginal epithelial cells (p > 0.05, Fig. 2b).

## Discussion

We demonstrate that several vaginal lubricant and moisturizing products commonly used by postmenopausal women inhibit both uropathogenic *E. coli* and commensal *Lactobacillus* spp. in an *in vitro* co-culture model without vaginal epithelial cells. However, when human vaginal epithelial cells were included in the co-culture, the products demonstrated less inhibition of *E. coli*. None of the products inhibited the growth of *Lactobacillus* spp. at a 1:100 dilution, regardless of whether immortalized human vaginal epithelial cells were added to the model.

Our results expand on previous *in vitro* studies which showed that KY Jelly is bactericidal to *L. crispatus* and *L. jensenii*^15^. Inhibitory effects of vaginal lubricants could be due to several of the ingredients, including glycerin, parabens, sorbic acid, chlorhexidine gluconate, all of which have been shown to have antimicrobial properties. Previously, parabens in mouthwash and vaginal moisturizer have been found to decrease or inhibit oral *Lactobacillus* spp.^16^. We found that Replens Long-Lasting moisturizer significantly inhibited *L. crispatus* growth while Replens Silky Smooth increased growth. Replens Silky Smooth is marketed as a preservative free lubricant and does not contain parabens, while the Replens Long-Lasting contains methylparaben. Both formulations of Replens, and all products except coconut oil inhibited *E. coli*, suggesting that *L. crispatus* may be less susceptible than *E. coli* to antimicrobial effects of these products.

When we added cultured, immortalized vaginal epithelial cells to the model, we demonstrated less inhibition of *E. coli* by the products that had been most inhibitory in the co-culture without vaginal cells. We used a 1:100 dilution of products to maintain viability of human cells, but even at this dilution *E. coli* growth was inhibited by all three products in the absence of human cells. One possible explanation for the difference in *E. coli* inhibition in the presence of cells involves the ability of bacteria to adhere to and proliferate on epithelial cell surfaces^17^. Not only does adherence potentially permit bacterial proliferation over that seen in liquid media, bacterial microcolonization of epithelial cell surfaces may also allow bacteria to access additional food sources. An inspection of the surface of bovine intestinal epithelial cells showed that adherent bacteria were digesting sloughed dead epithelial cells, suggesting another resource for bacterial survival and proliferation^18^. At the 1:100 dilution no products inhibited the growth of *Lactobacillus* spp., thus we were unable to assess how the presence of human vaginal epithelial cells modified lubricant inhibition of *Lactobacillus* spp.

Our data add to the existing literature showing that vaginal products and their ingredients affect bacteria growth and viability *in vitro*, but conclusive literature on effects *in vivo* is lacking. The majority of literature on the safety of lubricant products concerns HIV acquisition and transmission and the impact of these products on the vaginal epithelial barrier and mucosal inflammation, rather than uropathogens or commensal vaginal bacteria. A prospective study of 331 women found spermicide use correlated with increased vaginal colonization with *E. coli*, which in turn was associated with absent lactobacilli^19^. However, vaginal swabs from 235 women in a randomized trial of various nonoxynol-9 spermicide formulations found no significant alterations in vaginal microbiota with repeated use^20^.

We are unaware of any studies specifically examining the effect of vaginal products on factors that affect an individual’s risk of UTI. While it is reassuring that these products have a uniformly negative effect on uropathogenic *E. coli in vitro*, the abrogation of this effect in the presence of human vaginal epithelial cells suggests that the interaction may not be that simple. While the co-culture experiments that do not include human cells use a concentration of products that is more akin to what might be seen in actual use, the more dilute concentrations necessary to maintain human cells do inhibit E. coli when human cells are not present. This suggests factors in addition to concentration of product may contribute to bacterial survival or growth. In addition, the negative impact of some vaginal products on *Lactobacillus* spp. is worrisome.

Our study is limited by the lack of a better human vaginal model. The 1:100 product dilution needed to allow vaginal epithelial viability is not realistically translated to clinical use. In addition, the viscous properties of lubricants and moisturizers introduce additional variation to the results between technical replicates as well as between experiments. We did not assess mechanisms for the inhibitory effects, which could range from chemical toxicity to pH to osmolality. Strengths of this study include the addition of several clinical strains of uropathogenic *E. coli* taken from clinical urine cultures of women with acute cystitis, demonstrating equivalence between clinical and laboratory strains. The addition of vaginal epithelial cells brings the co-culture model closer to a human vaginal model and the differing results for *E. coli* demonstrate the potential drastic difference between *in vitro* and *in vivo* studies. We only tested the effects of products on *L. crispatus*, which is one of the most common vaginal species, and is being tested as a potential live biotherapeutic. However, results may not be generalizable to other vaginal *Lactobacillus* spp.

This study adds to the limited understanding of how common vaginal products and their ingredients may alter commensal and uropathogenic bacteria, a dynamic that is important in the acquisition and prevention of UTI. Although lubricants, moisturizers, and pH balancers are all used for symptoms of vaginal atrophy and other genitourinary pathology, our findings suggest that they may have varying effects on the urogenital microbiota and therefore on a woman’s risk for UTI.

## Materials and Methods

### Bacterial culture preparation

The bacterial species used in these experiments include a laboratory strain of *E. coli* (ATCC 8739) and 6 clinical strains of *E. coli* provided by the Massachusetts General Hospital microbiology lab, which had been isolated from women with cystitis. The Institutional Review Board at Massachusetts General Hospital has determined that this work does not meet the definition of human subjects’ research. The following reagent was obtained through the NIH Biodefense and Emerging Infections Research Resources Repository, NIAID, NIH: *L. crispatus*, Strain JV-V01, HM-103. Bacterial suspensions of *E. coli* were created by inoculating BD Difco Nutrient Broth (Fisher Scientific, MA) and incubating for 6 hours at 37 °C with agitation. For the *L. crispatus* suspensions, deMan, Rogosa Sharpe broth (MRS, BD Difco, Fisher Scientific, MA) was inoculated and incubated for 12 hours at 37 °C in an anaerobic chamber without agitation.

### Vaginal products

To achieve a lower viscosity for pipetting, Trimo-San and Replens Long-Lasting moisturizer were diluted 1:2 with sterile PBS. The following lubricants were used undiluted: KY Jelly, Replens Silky Smooth lubricant, generic coconut oil. The following solutions were prepared as positive controls (i.e. expected to inhibit bacterial growth) diluted in sterile PBS: 50 mM racemic lactic acid and methylparaben (Sigma-Aldrich, MA), reconstituted to 1% in ethanol. Two negative control conditions were used: sterile phosphate buffered saline (PBS, Thermo Fisher Scientific, UK) and diluted ethanol to match the ethanol content in the methylparaben condition (Table 1).

### *E. coli* co-culture

In a 96-well microplate, the following were added to each well: 75 µL fresh nutrient broth, 50 µL test product, 25 µL *E. coli* broth culture at 0.5 OD, adapted from Kalyoussef *et al*.^21^. Each of the seven *E. coli* strains were tested against the five products and four controls (Table 1). Corresponding control wells, which served as blanks in calculations of optical density at 600 nm (OD_600_), were created for every test condition containing 25 µL of nutrient broth medium instead of 25 µL of bacterial broth culture. Plates were incubated at 37 °C with agitation for 6 hours. OD_600_ measurements were taken at every 2 hours with a Biotek Gen5 plate reader (Burlington, VT).

### *Lactobacillus* spp. co-culture

In a 48-well microplate, the following were added: 400 µL fresh MRS media, 200 µL test product, 200 µL *Lactobacillus* spp. broth culture at 0.5 OD. *Lactobacillus crispatus* was tested against the five products and four controls (Table 1). The plates were incubated anaerobically at 37 °C without agitation for 12 hours. Supernatants were serially diluted and plated on MRS agar to count colony forming units (CFU) at both the start and end of incubation. After initial experiments to ensure that OD and CFU were measuring comparable phenomena (Supplemental Fig. 3), we continued with OD only for E. coli. However, for lactobacilli, OD results were very different from CFU results due to significant clumping of bacteria in the presence of lubricants, thus only CFU were used.

For both the *E. coli* and *L. crispatus* co-cultures, all experiments were performed at least twice, with each biological replicate containing technical triplicates for each condition, including blank control conditions. All ingredients were plated on agar to confirm viability of the broth pre-cultures and sterility of test materials. At the conclusion of the experiment, co-culture broth and negative controls were plated to confirm no contamination during experiments.

### Vaginal epithelial cell co-culture with vaginal product and bacteria

To test the observed interactions in a more high-fidelity model, the vaginal products with the most significant inhibitory effects in simple co-culture were then co-cultured with bacteria in the presence of human vaginal epithelial cells. Bacterial broths were prepared as described previously, and then centrifuged at 1000 RPM for 5 minutes. The bacteria pellet was re-suspended in Gibco keratinocyte serum free media (KSF, Fisher Scientific, MA) (supplemented with epidermal growth factor, bovine pituitary extract and CaCl_2_ per manufacturer’s instructions) to an OD_600_ of 0.15.

A 96-well tissue culture treated microplate was seeded with 4 × 10^5^ cells per well of an immortalized vaginal epithelial cell line (VK2, ATCC, VA) in KSF. Vaginal epithelial cells were incubated overnight to achieve >90% confluence. The model was first tested with different dilutions of selected vaginal products alone to choose the highest concentration compatible with epithelial cell survival. Cell death was assessed with a lactate dehydrogenase cytotoxicity assay (Fisher Scientific, MA). As in previous studies, a dilution of 1:100 was found to be the optimal dilution to avoid toxicity to human cells^15^.

After a 24-hour equilibration to allow vaginal cells to adhere to the plate and become confluent, 25 µL of the laboratory strain *E. coli* suspended in KSF were added to the vaginal epithelial cells with 75 µL of fresh KSF media and incubated for 1 hour. Then 50 µL of KY Jelly, Replens Long-Lasting moisturizer, or Trimo-San were added, diluted 1:100 with KSF cell culture medium. The co-culture was incubated at 37 °C, 2 hours aerobically with agitation for *E. coli*. At the conclusion of the co-culture, the bottoms of the wells were scraped with a pipette tip to release the adherent human cells. The entire well contents (cells, bacteria, medium) were serially diluted and plated to count CFU for each bacterium. For all vaginal epithelial cell co-cultures, KSF cell culture media was used as a negative control, and at least two experiments were performed for each condition, with technical triplicates of each condition. The experiment was simultaneously performed without vaginal cells, using 1:100 diluted products, KSF medium and bacterial suspension.

Similarly, the vaginal epithelial cells were separately co-cultured with *L. crispatus*, though in anaerobic conditions for 1 hour prior to addition of experimental substance. However, because the growth of the *Lactobacillus* spp. was inhibited in the KSF media alone, a 1:1 mixture of MRS/KSF medium was utilized for that portion of the experiment, which was found to have a minimal impact on human cell viability (Supplemental Fig. 4).

### Statistical analysis

Growth of each bacterial strain or species was determined by change in OD_600_ or CFU from the first baseline OD measurement (Supplemental Table 1) or a starting CFU/mL count of the *L. crispatus* innoculum, respectively. Using GraphPad Prism 6.0, the change in OD_600_ or CFU was compared between vaginal products and control with unpaired t-tests or ANOVA, with adjustment for multiple comparisons as appropriate. Statistical significance was set at p < 0.05.

## Supplementary information

Supplementary information.
