# Supplementary material for: Effect of commercial vaginal products on the growth of uropathogenic and commensal vaginal bacteria
Source: Sci Rep. 2020 May 6;10:7625. doi: 10.1038/s41598-020-63652-x (PMC7203152; doi:10.1038/s41598-020-63652-x)

Effect of commercial vaginal products on the growth of uropathogenic and commensal vaginal bacteria

Kristin J. HUNG, MD[1,2], Patricia L. HUDSON, MD[1,3], Agnes BERGERAT, PhD[3], Helai HESHAM, MD[1,3], Namit CHOKSI, MD, MPH, MMSc[3], Caroline MITCHELL, MD, MPH[3*]

Supplemental Material

Supplementary Table 1: Values for OD_{600} of the starting experimental mixture at Time 0 for the laboratory strain of E. coli. Each experiment included 3 technical replicates, which are averaged to give the starting value for each experiment for the purposes of this table (however, experimentally, each replicate was compared to itself to calculate the change in OD_{600}).

	Experiment		
Agent	1	2	3
Saline	0.042	0.175	0.072
KY Jelly 1:1	0.063	0.050	0.065
TrimoSan 1:2	0.053	0.135	0.078
Replens-LL 1:2	0.078	0.115	0.091
Replens-SS	0.033	0.138	0.112
Coconut Oil 1:1	0.055	0.078	0.086
Lactic acid 50mM	0.075	0.078	0.089
1:10 Methylparaben in EtOH 70%	0.067	0.082	0.090
1:10 Ethanol 70%	0.041	0.081	0.085

Figure Legends:

Supplemental Figure 1 Growth curves for clinical *Escherichia coli* strains when exposed to vaginal products. Growth curves for all clinical strains of *E. coli* A-F over 6 hours (simple co-culture without vaginal epithelial cells), as measured by change in OD600, are similar for each product. In all strains but clinical strain D, which demonstrated overall poor growth, there is a significant inhibition by all products except coconut oil ($p < 0.02$)

Supplemental Figure 2. Measurement of vaginal epithelial (VK2) cell viability after 1 hour of exposure to a 1:10 or 1:100 dilution of vaginal products demonstrates that at 1:100 dilution all products have similar cell death (measured by LDH assay) vs. media (KSF) control, while at 1:10 Trimosan has significant toxicity.

Supplemental Figure 3. (A) When the pattern of *E. coli* growth in the presence of KY Jelly was measured by OD600 vs. counting colony forming units (CFU), very similar trajectories and differences were seen. (B) When the pattern of *L. crispatus* growth was measured by OD600 vs. CFU, suppression of growth was seen when measuring by OD600 but not by CFU. (C) When comparing fold change in quantity from start to end of the experiment, similar values were seen for *E. coli* using both measurement strategies, but not for *L. crispatus* (ANOVA, $p = 0.05$).

Supplemental Figure 4: Immortalized epithelial cells were cultured for 24 hours in KSF alone or a 1:1 mixture of MRS:KSF. Using an LDH assay for cell death, the proportion of dead cells was found to be significantly different between these two conditions ($p < 0.001$), but viability

remained ~80% in the mixture, which was used to facilitate growth of *Lactobacillus crispatus* to allow measurement of potential inhibition by lubricants when human cells were present.

Supplemental Figure 1

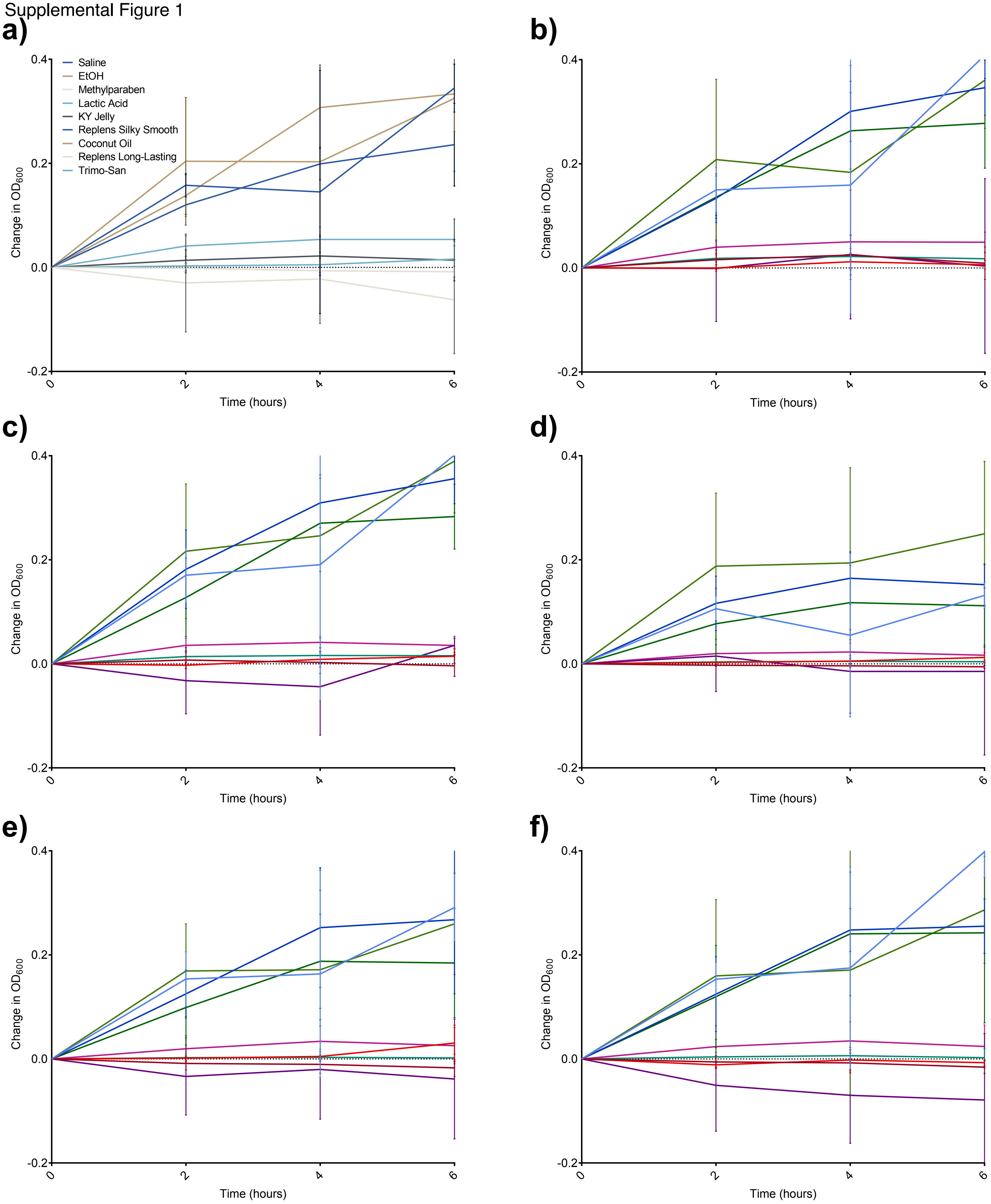

Supplemental Figure 2

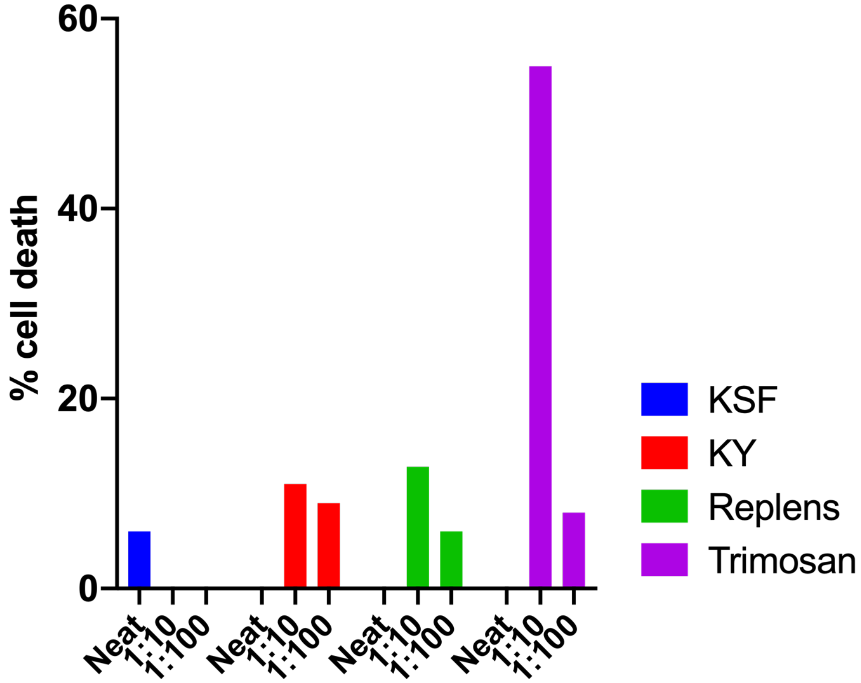

Supplemental Figure 3

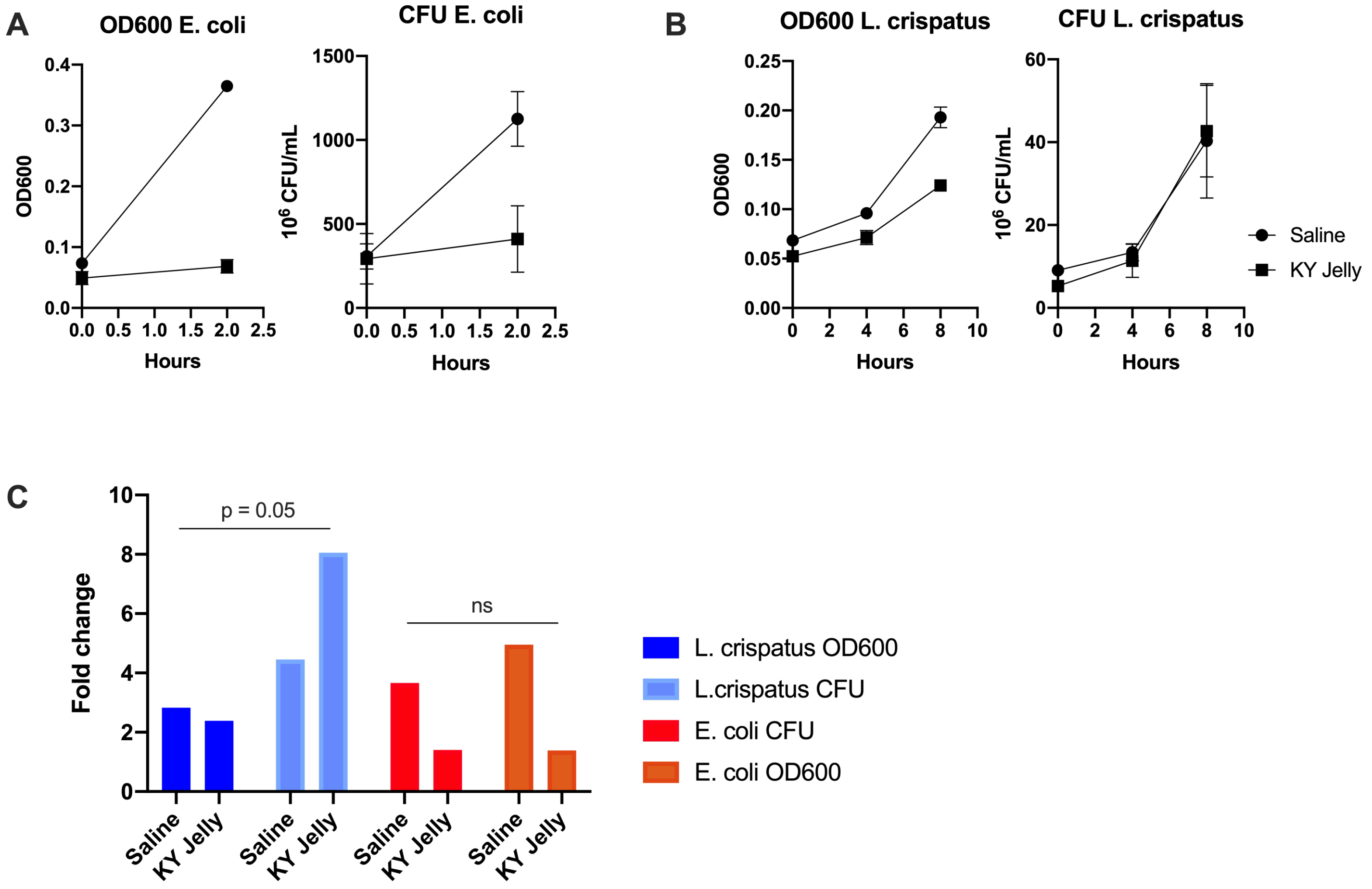

Supplemental Figure 4

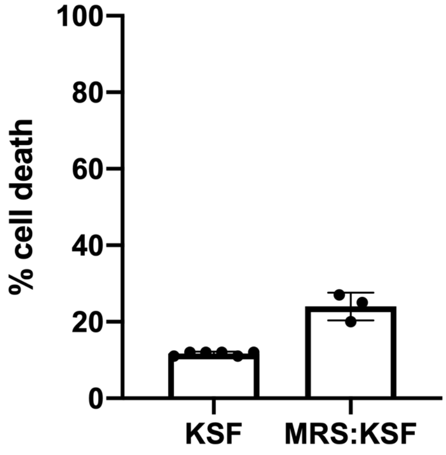

Supplement: Supplementary file 1 — Supplementary information. [file 41598_2020_63652_MOESM1_ESM.pdf]
